# Proapoptotic Effect and Molecular Docking Analysis of Curcumin–Resveratrol Hybrids in Colorectal Cancer Chemoprevention

**DOI:** 10.3390/molecules27113486

**Published:** 2022-05-28

**Authors:** Gustavo Moreno-Q, Angie Herrera-R, Andres F. Yepes, Tonny W. Naranjo, Wilson Cardona-G

**Affiliations:** 1 Chemistry of Colombian Plants Group, Institute of Chemistry, Faculty of Exact and Natural Sciences, University of Antioquia (UdeA), Calle 70 No. 52-21, Medellin 050010, Colombia; gustavo.morenoq@gmail.com (G.M.-Q.); andresf.yepes@udea.edu.co (A.F.Y.); 2 Medical and Experimental Mycology Group, Corporación para Investigaciones Biológicas, Medellin 050034, Colombia; tonny.naranjo@upb.edu.co; 3 School of Health Sciences, Pontifical Bolivarian University, Medellin 050034, Colombia

**Keywords:** curcumin, resveratrol, hybrid molecules, colorectal cancer, cell death, apoptosis, molecular docking

## Abstract

Different hybrids based on curcumin and resveratrol were previously synthesized and characterized by spectroscopic techniques. The most active molecules (**3a**, **3e**, **3i**, and **3k**) were evaluated in vitro as an approach to determine the possible mechanism of action of the hybrids. The results indicated that the evaluated curcumin/resveratrol hybrids induce mitochondrial instability in SW620 and SW480 cells. Moreover, these molecules caused a loss in membrane integrity, suggesting an apoptotic process mediated by caspases after the treatment with compounds **3i** (SW480) and **3k** (SW620). In addition, the results suggest that the mechanism of action of the hybrids could be independent of the p53 status. Furthermore, hybrids **3e** and **3i** caused G0/G1 phase arrest, which highlights the potential of these molecules not only as cytotoxic but also as cytostatic compounds. Hybrids **3e** and **3i** caused a negative modulation of the matrix metalloproteinase 7 (MMP7) on SW480 cells. These curcumin resveratrol hybrids could be potential candidates for further investigations in the search for potential chemopreventive agents, even in those cases with resistance to conventional chemotherapy because of the lack of p53 expression or function. Molecular docking simulations showed that compounds **3e**, **3i**, and **3k** bind efficiently to proapoptotic human caspases 3/7 proteins, as well as human MMP-7 and p53, which, in turn, could explain at the molecular level the in vitro cytotoxic effect of these compounds in SW480 and SW620 colon cancer cell lines.

## 1. Introduction

Despite colorectal cancer (CRC) being considered one of the most preventable cancers through modifications in lifestyle, it continues to be a leading cause of morbidity and mortality, being the third most incident malignancy with 1,931,590 new cases in 2020 and the second cause of cancer-related death worldwide counting about 935,173 deaths in the same year. Notwithstanding the enormous effort to progress in diagnosis, treatment, incidence, and mortality of CRC, it is unacceptably high [1,2]. By considering this important increase in the statistics and the widespread occurrence of the risk factors, extensive investigations are in progress to develop new therapeutic alternatives against CRC.

Current chemotherapy for CRC includes both single-agent and multiple-agent regimes, which are mainly based on 5-fluorouracil (5-FU) as the backbone of the treatment. This fluoropyrimidine is provided in combination with irinotecan (IRI), oxaliplatin (OX) complemented with folinic acid (leucovorin) as an adjuvant using the schemes named FOLFOX (5-FU + OX + leucovorin), FOLFIRI (5-FU + IRI + leucovorin), and FOLFOXIRI (5-FU + oxaliplatin + irinotecan + leucovorin). Other treatments use an alternative strategy composed of the oral form of 5-FU named capecitabine. These regimes include XELOX/CAPEOX (Capecitabine with oxaliplatin) and XELIRI/CAPIRI (Capecitabine with irinotecan) [3,4,5]. Although these schemes are effective, they induce different grades of toxicity, including neurological and gastrointestinal disorders and myelosuppression, among others, which leads to dose limitation and treatment discontinuation [3,6,7]. Despite all efforts to discover new therapies against cancer, it is still a leading cause of death worldwide.

Because of these facts, novel therapeutic approaches have been explored, including chemoprevention. It involves the use of external agents, either natural, synthetic, or biological, to prevent or suppress the development of a specific disease, including cancer, seeking to disturb the process of carcinogenesis [8,9,10]. The initial step in the discovery of novel drug candidates is based on the identification of hits and subsequent lead structures. Thus, different natural products have been used as important sources of bioactive compounds. In this sense, curcumin (CUR) and resveratrol (RES) have long been evaluated for exhibiting a wide spectrum of biological activities, including anticancer properties [11,12,13,14,15]. Although both curcumin and resveratrol alone exhibit great activity in vitro and in vivo, they have shown restricted clinical efficacy because of different factors, including low bioavailability and stability [16,17]. For this, they have been extensively studied to improve their pharmacological characteristics.

Considering these facts and knowing that many diseases are caused by defects in different biological targets, it is common that even the most promising hits will only influence one biological target, and it would probably not be sufficient to effectively combat multifactorial diseases such as cancer. Because of this, the strategy based on the use of molecules with different mechanisms is one of the methods adopted for the treatment of cancer [16,18]. In this sense, the synthesis of new molecules using curcumin and resveratrol as parental compounds has been evaluated.

Curcumin has been widely evaluated for exhibiting chemopreventive, antimetastatic, and anti-angiogenic potential. It has been investigated in different in vitro studies and animal models, and the results showed anticarcinogenic properties in inflammation-related and genetic CRC. Moreover, this compound has even been evaluated in humans as a supplement alone or in combination with conventional chemotherapy, but there is no clear evidence of the results because of the difficulties with the dosage, bioavailability, and optimal indications [19]. Similar findings have been reported for resveratrol. In vitro and in vivo studies have shown that this stilbene alone modulates different biomarkers, leading to the reduction of inflammation and prevention of tumorigenesis. It has even been evaluated in clinical trials, but the major obstacle reported is its poor bioavailability. Moreover, its efficacy is dependent on the type and stage of cancer, dosage levels, and treatment periods [20]. Considering these facts, designing new molecules based on these scaffolds is an important tool to potentiate their activity.

In this sense, the use of hybrid compounds has emerged as a promising strategy in medicinal chemistry and drug discovery research. This design is based on the covalent linking of two molecules with individual intrinsic pharmacological activity [21,22]. The combination of two pharmacophores with different biological activities, probably acting on different targets, could confer a dual activity to the molecule [23,24,25]. Thereon, the synthesis of hybrids based on curcumin/resveratrol (Cur/Res) has emerged as a promising strategy for the discovery of new therapeutic alternatives focused on cancer chemoprevention, seeking to improve the efficacy of each compound alone and the selectivity regarding conventional treatments [26,27,28,29]. 

**Scheme 1:**
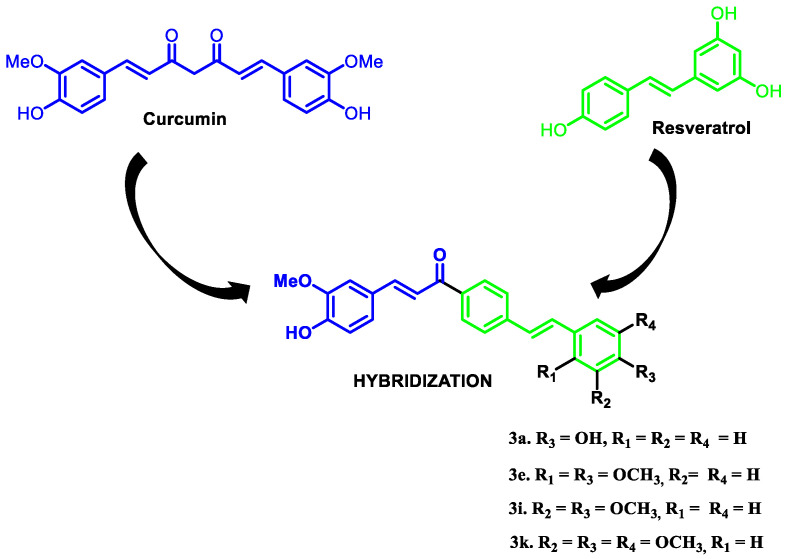
Design of hybrids based on curcumin and resveratrol. This includes a summary of the data taken from the previous publication from our research group [30].

In previous work, we reported the synthesis of Cur/Res hybrids together with the cytotoxic and antiproliferative activities in SW480 and SW620 colon carcinoma cells. The synthesis of Cur/Res hybrids, together with the full characterization and the whole experimental processes, was previously reported by our research group [30]. Scheme 1 shows the design of the obtained hybrids [30]. According to the results, hybrids **3e** and **3i** displayed the best activity and selectivity in SW480 cells. On the other hand, compounds **3a**, **3e**, and **3k** exhibited the best selective activity against SW620 cells, with IC_50_ values ranging from 11.52 ± 2.78 to 29.18 ± 4.36 µM for both cell lines after 48 h of treatment. We even found that hybrid molecules were more selective than the reference drug, 5-fluorouracil, the starting compound resveratrol, and the equimolar mixture of curcumin plus resveratrol. In addition, we reported good antiproliferative activity for these four hybrid molecules [30]. Considering those findings, we deepened the effect of these molecules on different mechanisms and targets and performed in silico studies of those hybrids to investigate the possible mechanism of action associated with these active molecules in colorectal cancer to identify potential candidates for further investigations in the search for potential chemopreventive agents.

## 2. Results and Discussion

### 2.1. Biological Activity

#### 2.1.1. Changes in Mitochondrial Membrane Potential (ΔΨm) and Plasma Membrane Integrity Induced by Curcumin/Resveratrol Hybrids

Mitochondrial membrane potential plays a pivotal role in apoptotic cell death [31]. In fact, it has been documented that those changes in ΔΨm occurred prior to caspase activation and cell death. It has been demonstrated that the opening of the mitochondrial permeability transition pore induces depolarization of the transmembrane potential conducive to the release of apoptogenic factors, which leads to caspase activation and apoptosis. For this, the assessment of mitochondrial depolarization has become the most used method for the identification of apoptotic cells. 

Considering the previous facts and the importance of the mitochondria in cell death, we evaluated if different curcumin–resveratrol hybrids could induce changes in ∆Ψm. A lipophilic dye (Dioc6(3)) selective for the mitochondria of live cells was used. This accumulates in the mitochondrial matrix. Moreover, after membrane depolarization (reduced ΔΨm), this dye is released into the cytosol. This mitochondrial staining is usually associated with the use of an exclusion dye such as propidium iodide (PI) to evaluate changes in the integrity of the cell membrane [32,33]. 

**Figure 1:**
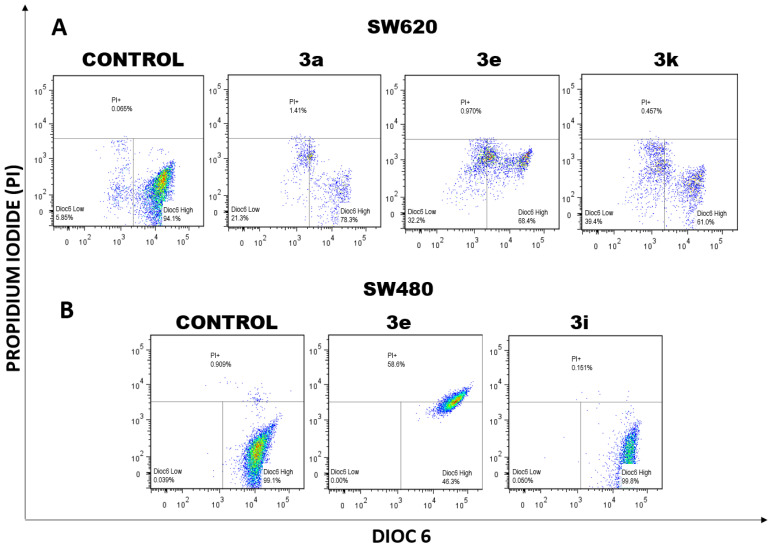
Changes in ∆Ψm and plasma membrane integrity induced by curcumin–resveratrol hybrids. Flow cytometry of cells stained with DiOC6 and PI. (**A**) SW620 cells. (**B**) SW480 cells. DiOC6 Low and PI+: cells in latency that lose membrane polarization and dead cells; DiOC6 High: live cells with high membrane polarization. One representative image is shown.

**Figure 2:**
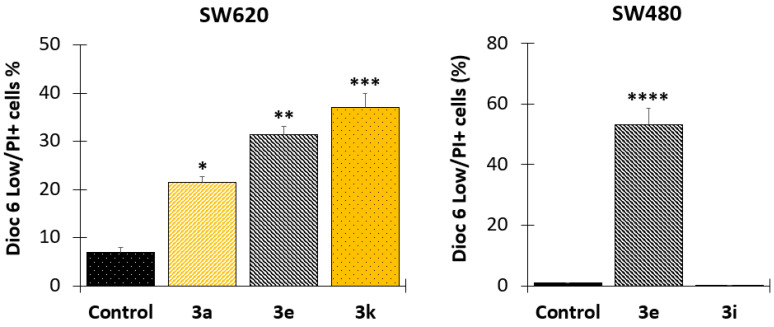
Representation of data in bar chart form. Cells in latency that lose membrane polarization and dead cells (DIOC6 Low and PI+). Data are presented as the mean ± SE of two independent experiments. *p* values lower than 0.05 were considered statistically significant (* *p* < 0.05; ** *p* < 0.01; *** *p* < 0.001; **** *p* < 0.0001). DMSO 1% (Control).

According to the results illustrated in Figure 1, mitochondrial stability of both SW620 (Figure 1A) and SW480 cells (Figure 1B) was clearly perturbed after the treatment with the hybrids, unlike the control. Compounds **3a**, **3e**, and **3k** caused significant changes in mitochondrial membrane potential of SW620 cells as evidenced by the increase in the population “Dioc6 Low” (left lower region) with subtle changes in the population with positive staining for PI (Figure 2), characteristics that are common in early apoptosis were cell membrane integrity are preserved [33]. On the other hand, hybrid **3e** caused changes in PI-positive cells, showing not only a loss in membrane polarization but also damage to the cell membrane, suggesting a process of death. 

Similar studies have been made with curcumin alone and its derivatives. Wang et al. (2021) [34] recently reported one molecule with potential activity in colon cancer cells (HCT116 and HT29), causing changes in mitochondrial membrane potential. In addition, Agarwal and colleagues (2018) [35] reported that the treatment with curcumin alone induced significant changes in the mitochondrial membrane potential of HT29 cells in a dose- and time-dependent manner. On the other hand, resveratrol (Res) has also been widely investigated in different cell models. Park and colleagues (2017) [36] evaluated a Res derivative in human hepatoma cells and reported that this molecule induced collapse in the mitochondrial membrane potential in HepG2 cells. Moreover, Delmas et al. (2003) [37] reported that resveratrol alone caused a decrease in ΔΨm, with a higher effect in SW480 cells, correlating with apoptosis. All these findings support the hypothesis that the evaluated hybrids probably induce cell death through the mitochondrial pathway.

#### 2.1.2. Effect of the Hybrids Based on Curcumin/Resveratrol on Cell Cycle Distribution

**Figure 3:**
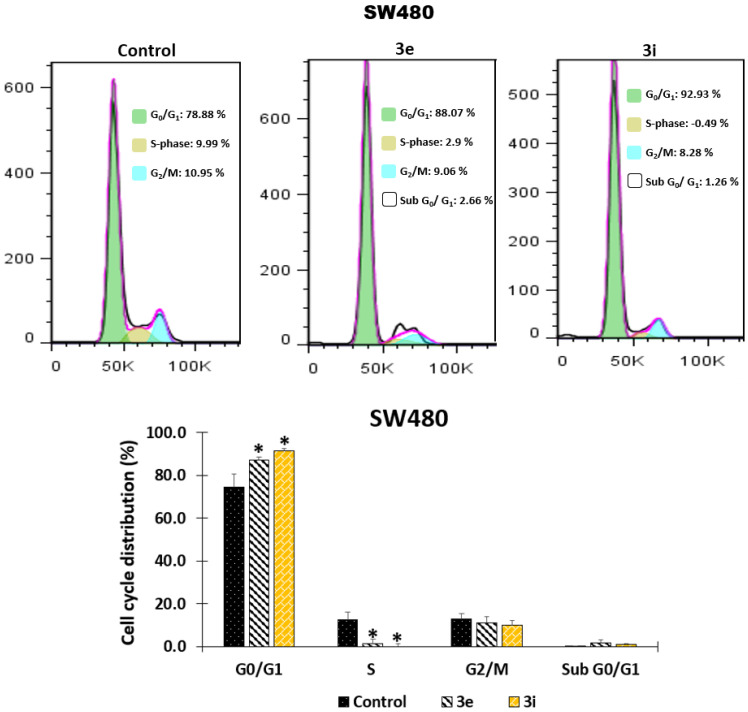
Cell cycle analysis of SW480 cells after treatment with DMSO (1%) or the hybrids based on curcumin/resveratrol. After 48 h of treatment, cells were labeled with PI and analyzed by DNA flow cytometry. The data indicate the percentage of cells in each phase of the cell cycle. All experiments were performed in duplicate and gave similar results. *p* values lower than 0.05 were considered statistically significant (* *p* < 0.05).

**Figure 4:**
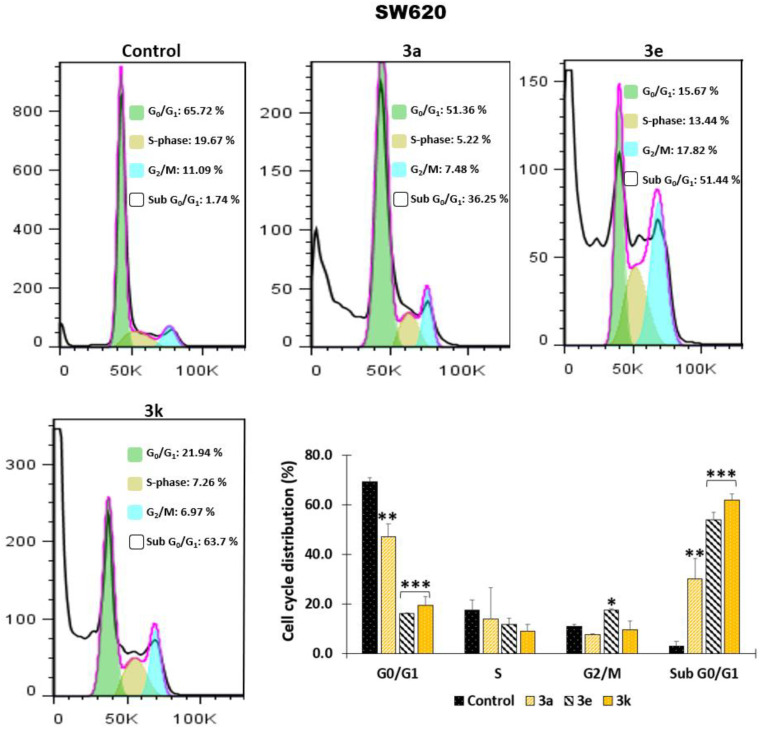
Cell cycle analysis of SW620 cells after treatment with DMSO (1%) or the hybrids based on curcumin/resveratrol. After 48 h of treatment, cells were labeled with PI and analyzed by DNA flow cytometry. The data indicate the percentage of cells in each phase of the cell cycle. All experiments were performed in duplicate and gave similar results. *p* values lower than 0.05 were considered statistically significant (* *p* < 0.05; ** *p* < 0.01; *** *p* < 0.001).

We were also interested in evaluating the effects of the hybrids based on curcumin/resveratrol on cell cycle progression in exponentially dividing cultures of SW480 and SW620 cells. Cells were treated with either DMSO alone (1%) or the hybrids at different concentrations depending on the IC_50_ values (the same concentration was used in all experiments). After 48 h of treatment, cells were labeled with PI and analyzed by DNA flow cytometry. A representative histogram for each cell line is shown in Figure 3 (SW480) and Figure 4 (SW620).

In SW480 cells (Figure 3), hybrids **3e** and **3i** caused a significant increase in cells in the G0/G1 phase with the corresponding decrease in cells in the S-phase. In addition, although there was a slight increase in the sub-G0/G1 population, these changes were not significant. On the contrary, the results observed on SW620 cells showed that more than half of 50% of the cells underwent death after 48 h of treatment with hybrids **3e** and **3k**. Moreover, compound **3a** exhibited more than 30% of cells in the death process, as detected in a prominent sub-G0/G1 peak (Figure 4). Furthermore, this increase in the population was consistent with a significant decline of the cells in the G0/G1 phase. 

Unlike this research, Agarwal et al. (2018) [35] previously reported that curcumin alone induces cell cycle arrest at the S phase and G2/M phase with subtle changes in G0/G1 phase using the human colon adenocarcinoma cell line HT29. In contrast, Wang and colleagues (2021) [36] recently reported identical findings, which agree with the results of this investigation. They evaluated a curcumin derivative and found that it causes a significant cell cycle arrest at the G1 phase with a decrease at the S phase and no changes at G2/M in human colorectal carcinoma cell line HCT116. On the other hand, different authors have also evaluated resveratrol alone and different derivatives of this. Schneider et al. (2000) [38] evaluated the effect of resveratrol on human colorectal carcinoma Caco-2 cells, and they found that a large proportion of cells accumulated in the S phase after 24 h of treatment. However, in the same study, the authors observed that 40 h post-treatment, the growth-inhibitory effect of resveratrol disappeared, and the cells restored a normal cell cycle. On the contrary, Colin et al. (2009) [39] found a time-dependent effect when evaluating different resveratrol analogs on SW480 cells for 24, 48, and 72 h. They reported that analogs induce cell cycle arrest in the early S phase with a decrease in the G1 phase and a virtually complete disappearance of the G2/M population. All these findings, together with the findings presented in the current publication, suggest that these modifications in the parental structures help to improve the efficacy of the compounds through time, causing a longer effect which probably makes them promising chemosensitizers for colorectal cancer therapy.

#### 2.1.3. Cell Death Induction by Curcumin/Resveratrol Hybrids

The plasma membrane has a pivotal role in homeostatic maintenance, acting as a direct barrier against the extracellular environment. This membrane contains protein sensors and receptors that allow signal transduction and transport of ligand molecules across the membrane. Considering these facts, the loss of membrane integrity puts an end to cellular life, and this event could be associated with different pathways of programmed cell death, including apoptosis, post-apoptotic secondary necrosis, and necroptosis, among others. 

**Figure 5:**
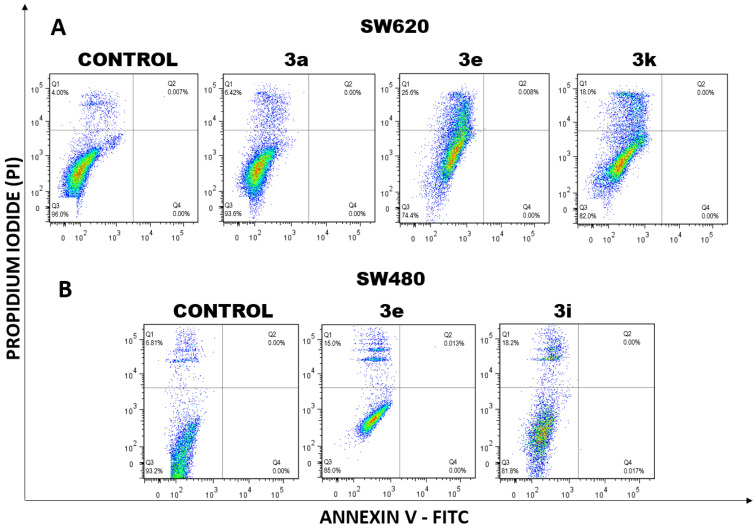
Apoptosis analysis of SW620 (**A**) and SW480 (**B**) cells in response to the treatment with curcumin/resveratrol hybrids. The histograms represent one of at least two different experiments. Q1 + Q2: Cells that lost membrane integrity (Late apoptotic cells, dead cells, necroptotic cells, secondary necrotic cells, others); Q3: Viable cells; Q4: Early apoptotic cells. Control cells were treated with DMSO (1%). All experiments were performed in duplicate and gave similar results.

**Figure 6:**
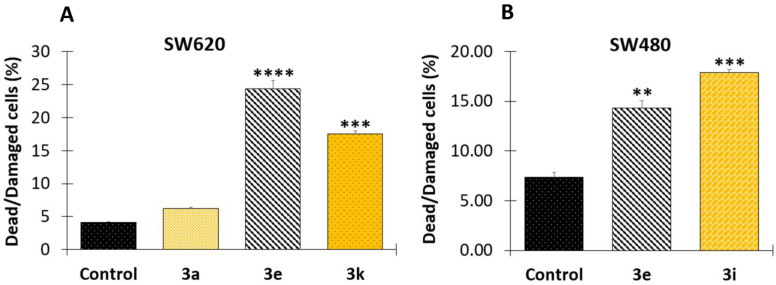
Representation of data in bar chart form. Dead and damaged cells which lost membrane integrity (Q1 + Q2). SW620 (**A**); SW480 (**B**). *p* values lower than 0.05 were considered statistically significant (** *p* < 0.01; *** *p* < 0.001; **** *p* < 0.001).

Supported by the previous findings observed for mitochondrial membrane potential and cell cycle distribution, we evaluated if the hybrids based on curcumin/resveratrol induce plasma membrane damage and possibly cell death. We used double staining with annexin-FITC and propidium iodide; the results are illustrated in Figure 5. After 48 h exposure, it was observed that hybrids **3a**, **3e**, and **3k** induce plasma membrane disintegration in SW620 (Figure 5A), and compounds **3e** and **3i** caused the same effect on SW480 cells (Figure 5B) as evidenced by the displacement of the cells through the upper quadrant with positive staining for propidium iodide (Figure 6A,B). 

Other authors have reported that the anticancer effect of curcumin on CRC cells is associated with the activation of the apoptosis pathway involving multiple molecular targets [9]. Similarly, different authors have found that resveratrol alone induces apoptosis in different cell lines of CRC [40]. Radhakrishnan et al. (2011) [41] found that resveratrol increased cell death of colon cancer cells after treatment with grape seed extract through an apoptotic pathway involving P53 and Bax:bcl-2 ratios. Juan and colleagues (2008) [42] reported that resveratrol induced apoptosis in HT-29 cells through the mitochondrial pathway triggered by the production of ROS. In addition, Park et al. (2007) [43] showed that resveratrol initiated endoplasmic stress, which eventually caused apoptotic death of HT-29 colorectal cancer cells. Considering all these findings together with the fact that losing membrane integrity puts an end to cellular life [44], we hypothesized that the Cur/Res hybrids evaluated induce a death process in SW480 and SW620 cells; however, it is necessary to carry out further studies to elucidate the exact signaling pathway related to the mechanism of action of these molecules.

#### 2.1.4. Determination of Apoptotic Biomarkers

**Figure 7:**
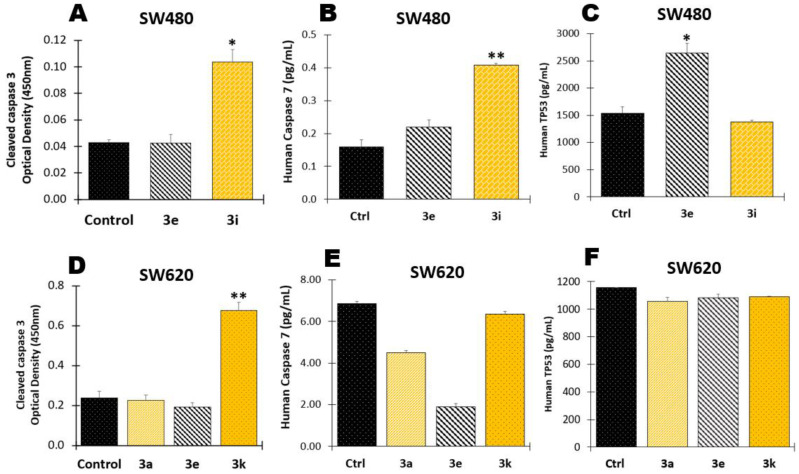
Determination of apoptotic biomarkers in SW480 (**A**–**C**) and SW620 (**D**–**F**) cells. Cells were harvested 48 h after treatment with hybrids **3a**–**3d** or the vehicle control (DMSO 1%). Protein levels of cleaved caspase3 (**A**,**D**), human caspase 7 (**B**,**E**) and human TP53 (**C**,**F**). Data are presented as the mean ± SE of two independent experiments. (* *p* < 0.05; ** *p* < 0.01). Optical density is directly proportional to the concentration of protein.

To find a better explanation for the previous results, different apoptotic biomarkers were included in this study. Firstly, considering that apoptosis is a highly regulated process, we evaluated caspases 3 and −7, the final executioners of the apoptotic pathway, which are responsible for initiating cell shrinkage, membrane blebbing, and DNA fragmentation, which are the hallmarks of the degradation phase of apoptosis [45]. According to the results, it was found that only compound **3i** induced a significant increase in the levels of both proteases in SW480 (Figure 7A,B). In addition, hybrid **3k** was the only one that caused significant changes in caspase 3 in SW620 cells (Figure 7D) without important modifications in caspase 7 (Figure 7E). These findings suggest the possible mechanism of action of hybrids **3i** and **3k** could be related to apoptosis. 

In addition, considering that the tumor-suppressor protein p53 has a pivotal role in the regulation of different cellular processes such as apoptosis [46,47], we evaluated if the Cur/Res hybrids could modulate the expression of this protein. According to the results, none of the compounds evaluated caused important changes in p53 in SW620 cells (Figure 7C,F); however, compound **3e** caused a significant increase in the concentration of the tumor suppressor protein in SW480 cells (Figure 7C). This is an important finding because p53 is mutated in SW480 and SW620 cells, and our experimental results suggest that hybrid **3e** could activate the protein in SW480 cells. This is consistent with the investigations reported by other authors who have demonstrated that p53 retains some of the functions maintaining residual DNA-binding ability [48], and it is possible to activate it both in vitro and in vivo through different mechanisms [48,49]. All these findings suggest that Cur/Res hybrids have the potential for further investigations to design molecules that could potentially be used as therapeutic alternatives or adjuvants in the treatment of colorectal cancer, even in those cases with resistance to conventional chemotherapy because of the lack of p53 expression or function. This could also be supported by those studies where curcumin alone has been used as an adjuvant regime to FOLFOX, improving overall survival in the patients [19,50].

These findings lead us to hypothesize that the apoptotic process induced by the Cur/Res hybrids could be mediated by caspases but independent of the p53 status. Similarly, Leischner and colleagues (2021) [51] evaluated resveratrol in different cell lines, including colon cancer, and they reported that the effect was predominantly independent of p53. Moreover, other studies have also demonstrated that curcumin induces apoptosis independent of p53 status in various cell lines, including colon carcinoma, involving oxidative stress, highlighting the therapeutic potential of these scaffolds in the management of colon carcinoma, especially in those resistant to conventional chemotherapy due to defects in p53 expression or function [9,52]. In addition, it was found that the hybrids evaluated did not cause important changes in the levels of caspase 8 (data not shown), which suggests the apoptotic process is not mediated by extrinsic pathway, supporting our findings of the effect of these hybrid molecules on mitochondrial membrane potential.

#### 2.1.5. Effect of Curcumin/Resveratrol Hybrids in the Activity of Matrix Metalloproteinase 7 (MMP-7)

**Figure 8:**
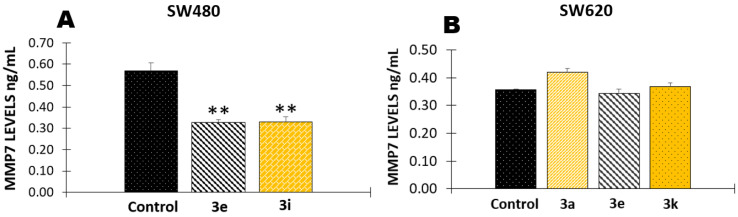
Concentration of matrix metalloproteinase 7 (MMP7) on SW480 (**A**) and SW620 cells (**B**). Data are presented as the mean ± SE of two independent experiments (** *p* < 0.01).

The extracellular matrix (ECM) provides structural and biochemical support for the cells, regulating signaling processes such as differentiation, adhesion, and invasion. Cancer cells interact with the ECM causing structural changes to facilitate migration. Different proteins such as matrix metalloproteinases (MMPs) are involved in the ECM remodeling and degradation, acting as proteolytic enzymes. Moreover, MMP7 has long been evaluated in colorectal cancer because of the correlation between an increase in this endopeptidase and CRC invasion [53,54]. For this reason, the authors evaluated if the most active hybrids based on curcumin/resveratrol could regulate the activity of this enzyme in SW480 and SW620 cells. According to the results, compounds **3e** and **3i** induced significant negative modulation of MMP7 in SW480 cells (Figure 8A), but none of the compounds evaluated caused changes in SW620 cells (Figure 8B). This finding suggests that the evaluated hybrids could avoid cell migration; however, further studies are needed to confirm these results.

### 2.2. Docking Studies and Prediction of Binding Pose

Aiming to investigate the binding mechanism at a molecular level to present a reasonable explanation by which hybrids **3e**, **3i**, and **3k** induced cell death in SW480 and SW620 cells, docking-based protocols were performed. From the experimental findings, targeting proapoptotic human caspases 3/7 proteins, as well as by down-regulating the expression levels in human MMP-7 and p53, might be responsible for the caused in vitro cytotoxic effect of **3e**, **3i**, and **3k** in SW480 and SW620 colon carcinoma cell lines. According to biological assays, it was demonstrated that compounds **3i** and **3k** had a considerable modulation of caspase 3, whereas **3i** also showed a remarkable inhibitory effect in caspase-7 and MMP-7. Moreover, compound **3e** was active not only in MMP-7 but also in the modulation of the mutant p53. In this respect, these compounds were docked into each catalytic domain of human X-ray crystallographic structures of caspase 3/7, MMP-7, and p53 proteins via grid-based ligand docking with AutoDock Vina and affinity scores along with the best binding pose and protein–ligand interactions were examined (Table 1).

For caspase 3/7, first, the AutoDock Vina protocol inside the caspase 3/7 binding domain was validated through self-docking. We performed a comparison between the crystallographic binding mode deposited in PDB and the re-dock of the Ac-DEVD-CMK (red) ligand inhibitor. In order to carry out this validation, the root-mean-square deviation (RMSD) value was calculated to correlate the differences between the atomic distances. As illustrated in Figure 6A, the docked conformation predicted for Ac-DEVD-CMK (in *cyan) is spatially close to the crystallographic structure pose (in red), obtaining an average RMSD value of 1.075 Å. In addition, the best binding energy calculated for Ac-DEVD-CMK was estimated at about −8.2 kcal/mol. These findings indicate a high level of feasibility in our protein–ligand docking protocol, which was able to reproduce the binding pose of the co-crystallized ligand in caspase 3/7 deposited in the PDB ID: 5i9b [57] and PDB ID: 1f1j [51], respectively. 

**Figure 9:**
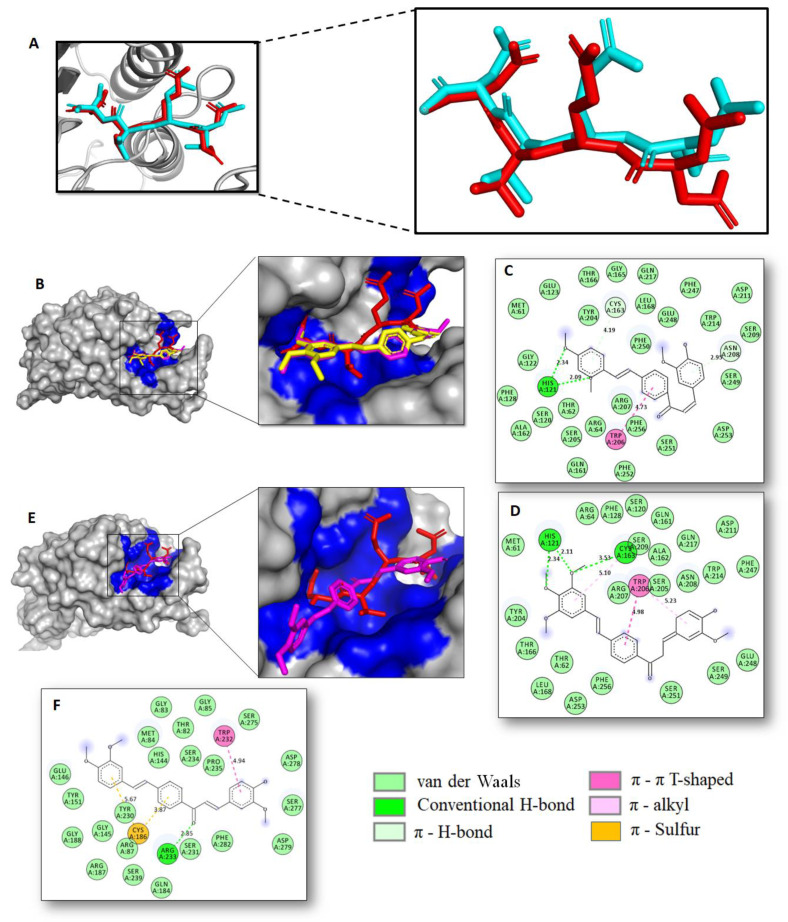
(**A**) Self-docking validation. Alignment of the best-docked pose of Ac-DEVD-CMK (in cyan) and the crystallographic binding mode (in red). (**B**) Superposition of the best docked conformations of hybrids **3i** (magenta) and **3k** (yellow), and crystallographic binding mode of inhibitor Ac-DEVD-CMK (red) within the caspase-3 active site. (**C**,**D**) 2D protein–ligand plot interaction for **3i** and **3k** into the caspase-3 domain. (**E**) Superposition of the best docked conformation of hybrid **3i** (magenta) and crystallographic binding mode of inhibitor Ac-DEVD-CHO (red) within the caspase-7 active pocket. (**F**) 2D protein–ligand plot interaction for **3i** into the caspase-7 active site. Catalytic residues are colored in blue.

After the docking protocol was validated, an exhaustive search in the binding pocket was carried out in order to establish key binding site points when hybrids **3i** and **3k** (magenta) were docked into the caspase 3/7 catalytic domains. Docking results showed that compounds **3i** (magenta) and **3k** (in yellow) display a good binding affinity for human caspase-3 (PDB ID: 5I9B) with good binding free energy close to −8.5 kcal/mol, fitting well in the active cavity of caspase-3, as illustrated in Figure 9B, with docking conformations that were in good agreement with the X-ray crystallographic pose of the peptide-based inhibitor Ac-DEVD-CMK (red). These particular facts support our proposal: **3i** and **3k** might block caspase-3 function with good binding affinity preventing cell growth and proliferation. In addition to this consideration, a visual examination of the 2D protein/ligand interaction plot (Figure 9C,D) also could support these preliminary findings. Thus, our analysis revealed critical hydrogen bonds and hydrophobic interactions formed between compounds and caspase-3. The **3i** and **3k** formed two conventional H-bonds with His121 (N-H···O, 2.09 Å (137.88°) and 2.34 Å (143.63°); 2.11 (144.98°) and 2.34 Å (133.35°)), respectively) and one π-π stacking contact with Trp206 (π···π, 4.44 Å (for **3c**) and 4.99 Å (for **3d**)), which have been identified as vital for caspase-3 function [57]. Moreover, **3k** also interacts with caspase-3 through one additional H-bond with key Cys163 (S-H···O, 3.53 Å, 110.70°). The molecules also formed diverse hydrophobic interactions, which would play a key role in the stabilization during the binding event. 

On another side, docking action for the most active compound **3i** within the active site of the caspase-7 protein target (PDB ID: 1f1j) revealed that this compound is well accommodated into the pocket domain of the enzyme with a good binding affinity of about −7.8 kcal/mol, showing a docked conformation in good agreement with the X-ray crystallographic pose of the peptide-based inhibitor Ac-DEVD-CHO (red), as illustrated in Figure 9E. This finding supports our proposal that **3i** could prevent cell growth and proliferation by modulating the caspase-7 function. This last statement was also supported by a rigorous visual examination of the 2D protein/ligand interaction plot after the docking event (Figure 9F), which would indicate that **3i** binds to caspase-7 by forming several interactions with those key amino acid residues: one strong hydrogen bond interaction with Arg233 (N-H···O, 2.85 Å, 104.99°), one π-sulfur contact with Cys186, one π-π interaction with Trp232 (π···π, 5.17 Å), as well as numerous hydrophobic contacts which may help to stabilize its conformation during the binding occurrence. Finally, we noted that **3i** not only was well accommodated inside the binding cavity of caspase-7 but registered a ligand interaction pattern that was consistent with the binding interaction pattern of the enzyme-inhibitor–substrate complex deposited in the PDB ID: 1f1j [58].

Next, to provide a probable explanation by which hybrids **3e** and **3i** regulate the in vitro expression of the human MMP-7 in SW480 cells, we performed protein–ligand docking simulations to examine potential binding modes of these compounds with MMP-7. Matrilysin or matrix metalloproteinase 7 (MMP7) is a zinc-dependent endopeptidase that is thought to be involved in the adhesion of cancer cells and plays an important role in tumor metastasis with high significance in colorectal cancer [55]. 

**Figure 10:**
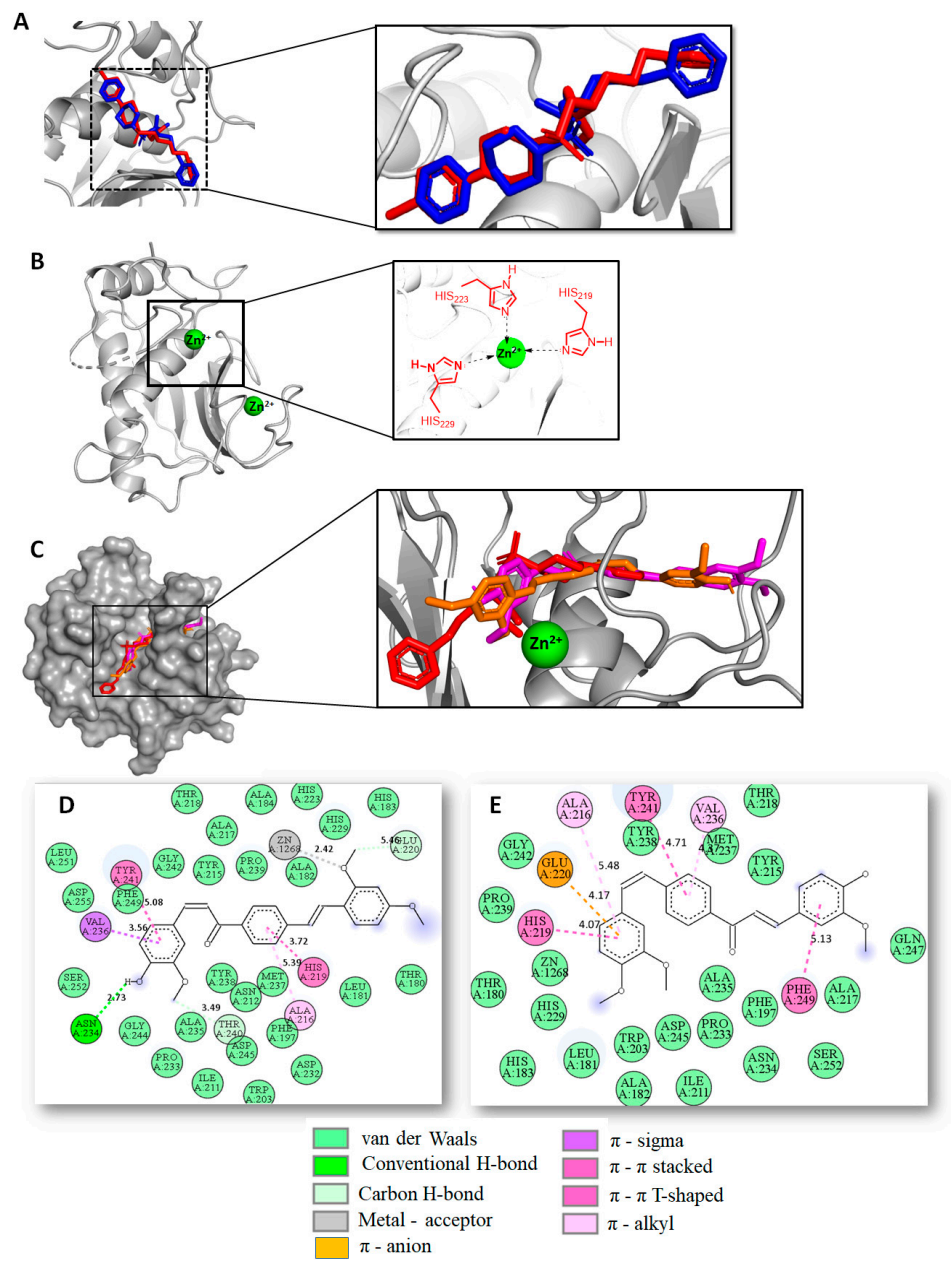
(**A**) A. Self-docking validation. Alignment of the best-docked pose of the hydroxamate ligand inhibitor (HLI) (in blue) and the crystallographic binding mode (in red). (**B**) Histidine triad chelates zinc into the catalytic domain of MMP-7. The catalytic zinc is represented by a green sphere. (**C**) Superposition of the best binding modes of hybrids **3e** (orange) and **3i** (magenta), and crystallographic binding mode of co-crystalized hydroxamate MMP-7 inhibitor (red) within the MMP-7 catalytic site. (**D**,**E**) 2D protein–ligand plot interaction for **3e** and **3i** into the MMP-7 active domain.

In this light, docking experiments start with the ligands located in the MMP-7 zinc-catalytic domain and closed to three histidines coordinate Zn^2+^. First, aiming to accomplish high throughput, the AutoDock Vina protocol inside the MMP-7 zinc-catalytic domain was validated through self-docking. We performed a comparison between the crystallographic binding mode deposited in PDB and the re-dock of hydroxamate ligand inhibitor (HLI). In order to carry out this validation, the root-mean-square deviation (RMSD) value was calculated to correlate the differences between the atomic distances. As illustrated in Figure 10A, the docked conformation predicted for HLI (blue) is spatially close to the crystallographic structure pose (red) with an optimal RMSD value of 1.811 Å. In addition, the best binding energy calculated for HLI was estimated at −8.3 kcal/mol. These findings indicate a high level of feasibility in our protein–ligand docking protocol, which was able to reproduce the binding pose of the co-crystallized ligand deposited in the PDB ID: 2y6d.

After the docking protocol is validated, an exhaustive search in the binding pocket was carried out in order to establish key binding site points when hybrids **3e** (orange) and **3i** (magenta) were docked into the MMP-7 zinc-catalytic domain. Molecular docking results suggested that **3e** and **3i** bind to MMP-7 with high affinity in about −10.3 kcal/mol, fitting well within the catalytic domain binding directly to the catalytic zinc ion (Figure 10B,C), as well with those three histidine residues (His219, His223, and His229) ligated to the catalytic zinc site. In general, docking studies showed several simultaneous interactions between MMP-7 and **3e** and **3i**, respectively, including metal-ion interaction, H-bonds, π-contacts, and multiple hydrophobic bonding interactions, which take place along the Zn-catalytic site (Figure 10D,E). In particular, **3e** binds to MMP-7 protein by forming one metal ion interaction between the catalytic Zn2+ ion and the oxygen atom of the methoxy group present in the stilbene moiety (Zn···O, 2.42 Å). One conventional hydrogen bond with Asn234 (O-H···H, 2.73 Å, 124.22°), two π-π interactions with His219 (π···π, 3.72 Å) and Tyr241 (π···π, 5.08 Å), as well as one π-σ contact with Val236 (π···σ, 3.56 Å) and one π-alkyl interaction with Ala216 (C-H···π, 5.39 Å) were also observed during the interaction of the compound **3e** with the MMP-7 protein. On the other hand, **3c** formed three π-contacts with His219 (π···π, 4.07 Å), Tyr241 (π···π, 4.71 Å), and Phe249 (π···π, 5.13 Å), as well as one π-anion interaction with the negatively charged Glu220 (π···π, 4.17 Å). It was also found that **3e** formed two hydrophobic π-alkyl contacts at the residue position Ala216 (C-H···π, 5.48 Å) and Val236 (C-H···π, 4.37 Å). The ligand-protein docking results for **3e** and **3i** showed that additional interactions, particularly hydrophobic contacts, were also found, which would play an important role in the stabilization of protein–ligand complexes. It is worth mentioning that **3e** and **3i** bonded to MMP-7 with a similar binding interaction pattern to the HLI–MMP7 complex deposited in the PDB ID: 2y6d. This computational evidence indicates that these compounds could modulate the MMP-7 catalytic activity by blocking MMP-7 access to the catalytic Zn site, therefore preventing cell growth and proliferation in SW480 cells. 

**Figure 11:**
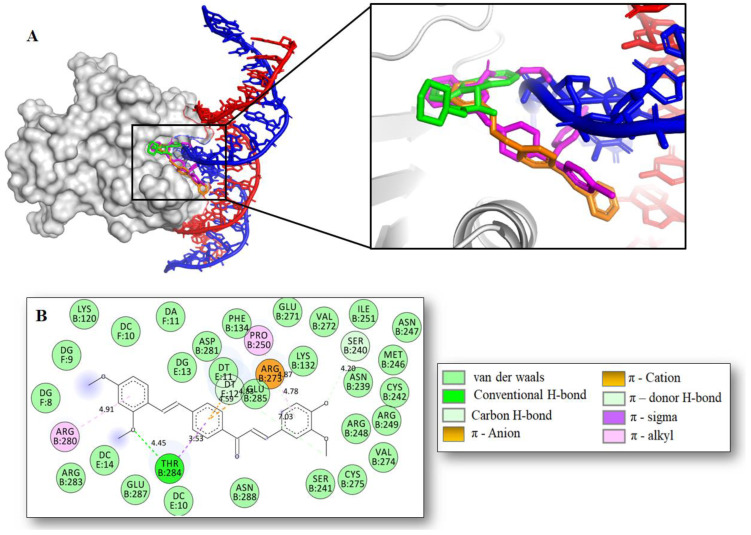
(**A**) Superposition of the best docked conformation of p53-modulators SCH529074 (magenta) and NSC194598 (green) and **3e** (orange) into the open L1/S3 pocket DNA-binding site of p53 protein. Double helix of DNA is represented in two-color (blue and red). (**B**) 2D protein–ligand plot interaction for **3e** into the p53 DNA-binding domain.

The tumor suppressor protein p53 is one of the most attractive targets in cancer drug development pipelines, which often appear dramatically altered in human cancer. Reactivating mutant p53 protein provides a valuable opportunity to combat cancer progression. In this paper, we experimentally found that compound **3e** caused a significant increase in the concentration of p53 in SW480 cells (Figure 7C), which, in turn, might indicate that the cytotoxic and antiproliferative response registered for **3e** in SW480 cells could also be associated with restoring or modulating p53 protein functions. These experimental results are in good agreement with previous work that demonstrated that curcumin would have the ability to bind to the p53-DNA-binding domain [56]. Aiming to understand at the molecular level how **3e** affects p53 protein, protein–ligand docking simulations were performed to examine potential binding modes of this compound to the crystal structure of the p53 core domain in complex with DNA (PDB ID: 1TSR). Likewise, two potent quinazoline-based p53-DNA-binding inhibitors, SCH529074 and NSC194598, were also evaluated in this study. The DNA-binding domain of p53 is constituted by ten hot spot amino acid residues Arg273, Arg248, Thr284, Glu285, Pro250, Val272, Lys164, Lys132, Ser241, and Ser240, which are critical for the biological activity of p53 [56]. To carry out docking experiments, **3e** was therefore positioned between the H2 helix and L3 loop of p53 protein harboring DNA-contact residues as a druggable binding pocket. Our results showed a considerable binding affinity of **3e** (−7.3 kcal/mol) within the DNA-binding site of p53, which was comparable to those of inhibitors SCH529074 (−7.0 kcal/mol) and NSC194598 (−7.3 kcal/mol). Moreover, both **3e** and inhibitors fit well within the DNA-binding pocket, as illustrated in Figure 11A. We found that **3e** binds to p53 protein through several intermolecular interactions (Figure 11B) that include both one conventional hydrogen bond (O-H···O, 4.45 Å, 148.61°) and one π-σ interaction (3.53Å) with the critical Thr284 residue. It was also found that **3e** formed one π-cation contact with the positively charged Arg273 (4.83 Å) and two π-alkyl interactions at the residue position Pro250 (4.87 Å) and Arg280 (4.91 Å), while numerous hydrophobic interactions that potentially confer stability during the binding event were also registered. According to the docking evidence, the in vitro modulation of p53 caused by **3e** could be associated with the restoration of wild-type p53 function in tumor cells, which would be in good agreement with experimental measurements.

Altogether, the computational findings were in good concurrence with the in vitro biological measures, which has enabled us to infer that the cytotoxic and antiproliferative response for the tested hybrids in human colon cancer cell lines SW480 and SW620 could be related to the ability of these compounds to bind to multiple colorectal cancer-related targets.

## 3. Materials and Methods

### 3.1. Source of the Hybrid Molecules

All compounds evaluated in this study were synthesized in our research group named Chemistry of Colombian Plants group (Química de Plantas Colombianas) Faculty of Exact and Natural Sciences from the University of Antioquia (Medellin, Colombia). These were characterized by spectroscopic techniques such as nuclear magnetic resonance and high-resolution mass spectra. The full characterization of the synthesis, the cytotoxic, and antiproliferative activities of these hybrids was previously published by our research group [30]. 

### 3.2. In Vitro Biological Assays

#### 3.2.1. Cell Line Culture and Treatments

Two human tumor cells were used in this study: colon adenocarcinoma (SW480) and its metastatic derivative (SW620). Cell lines were obtained from The European Collection of Authenticated Cell Cultures (ECACC, Salisbury, UK). All cell lines were cultured as monolayers and maintained in Dulbecco’s Modified Eagle Medium, supplemented with 10% heat-inactivated (56 °C) horse serum, 1% penicillin/streptomycin, and 1% non-essential amino acids (Gibco Invitrogen, Carlsbad, CA, USA). For all experiments, horse serum was reduced to 3%, and the medium was supplemented with 10 mg/mL insulin, 5 mg/mL transferrin, and 5 ng/mL selenium (ITS-defined medium; Gibco, Invitrogen, Carlsbad, CA, USA). Cell cultures were incubated at 37 °C in a humified atmosphere with 5% CO_2_ [26]. Cells were allowed to grow for 24 h before treatment. After that, SW480 cells were exposed to hybrids **3e** and **3i**, using the IC_50_ values (29.18 ± 4.36; 11.52 ± 2.78, respectively), and the SW620 cell line was treated with compounds **3a**, **3e**, and **3k** with the IC_50_ (20.15 ± 1.21; 20.44 ± 3.51; 11.81 ± 1.20, respectively). All cells were treated for 48 h prior each test.

#### 3.2.2. Double Staining for Mitochondrial Membrane Potential (ΔΨm) and Plasma Membrane Integrity

The mitochondrial membrane potential was estimated using the double fluorescent staining with DiOC6(3) and PI. Briefly, after the treatment with the hybrids for 48 h, cells were harvested by scraping using the same culture media. Later, they were stained with DiOC6(3) (3,3′-dihexyloxacarbocyanine iodide, Thermo Fisher Scientific, Waltham, MA, USA) and propidium iodide (PI), incubating for 30 min in darkness, at room temperature. In order to quantify the cells, 10,000 events were analyzed by flow cytometry with excitation at 488 nm and detection of the emission with the green (530/15 nm) and the red (610/20 nm) filters [59,60].

#### 3.2.3. Effect of Hybrids Based on Curcumin/Resveratrol on Cell Cycle Distribution

Cell cycle analyses were carried out as follows: after treatment, cells were collected by scraping and centrifuging, and the cell pellet was resuspended with phosphate-buffered saline (PBS). The cell suspension was fixed with 1.8 mL of ethanol (70%) at 4 °C, for 1 h. Then, cells were washed twice by centrifugation, using PBS, resuspending the pellet in 300 µL of PBS containing 0.25 mg/mL RNAse (Type I-A, Sigma-Aldrich, Darmstadt, Germany) and 0.1 mg/mL PI. Samples were incubated in the dark at room temperature for 30 min. The PI fluorescence of 10,000 events was analyzed using a FACS Canto II flow cytometer (BD Biosciences, San Jose, CA, USA), with excitation at 488 nm and detection of the emission at 610 nm. Data were analyzed using the software FlowJo v. 7.6.2 (FlowJo, Ashland, OR, USA) [59,60].

#### 3.2.4. Cell Death Induction by Curcumin–Resveratrol Hybrids

Phosphatidylserine exposure and membrane damage were evaluated through double staining with Annexin-V-FLUOS and propidium iodide (Roche Diagnostics), following the manufacturer’s instructions. Changes were measured by flow cytometric analysis of SW480 and SW620 cells after 48 h of treatment with the curcumin/resveratrol hybrids. After treatment, cells were collected by scraping, and the pellet was washed. Subsequently, cells were resuspended in a solution with Annexin-V-FLUOS and PI and incubated for 20 min in darkness before the analysis by flow cytometry. Data were analyzed using the software FlowJo 7.6.2 (Ashland, OR, USA). Cells with single staining for Annexin V/FITC were considered early apoptotic cells. Cells with positive staining for PI were considered late apoptotic cells, dead cells, necroptotic cells, or secondary necrotic cells. Assays were performed in duplicate.

#### 3.2.5. Determination of Apoptotic Biomarkers

After the cells were treated for 48 h with the hybrids, they were collected by scraping and lysed with Cell Lysis Buffer (1X, Ref. #9803). The supernatant was used to determine the effect of the compounds on the modulation of different apoptotic biomarkers. The kit for detecting caspase-7 was obtained from Elabscience Biotechnology Co. (Wuhan, China); whereas cleaved caspase-3 and p53 were obtained from Cell-Signaling Technology (Danvers, MA, USA). The assays were performed according to the manufacturer’s instructions [61].

#### 3.2.6. Statistical Analysis

Data are reported as mean ± SE (standard error) of at least two independent experiments. Statistical significance between non-treated and treated cells was evaluated by one-way ANOVA followed by the Dunnett’s test (*p*  ≤  0.05). Data were analyzed with GraphPad Prism version 7.04 for Windows (Graph Pad Software, San Diego, CA, USA).

### 3.3. Computational Methods

The chemical structure of compounds **3e**, **3i**, **3k**, as well as the inhibitors Ac-DEVD-CMK, NSC194598, SCH529074, and hydroxamate MMP-7 inhibitor (HLI), were used as ligands in the computations approaches. Their 2D structures were drawn in ChemDraw 17.0 software (Cambridge Soft, Cambridge, MA, USA) and saved as MDL MoL files. The Chem3D 17.0 (Cambridge Soft, USA) was used to generate 3D structures of all ligands and energetically minimize them by the MM2 force field. The Discovery Studies Visualizer program was used to rewrite the data files into pdb format. AutodockTools were used to parameterize ligand structures to compute Gasteiger partial atomic charges and to add full hydrogens, as well as to assign rotatable bonds. The resulting structure was saved in the required format for use with AutoDock. Then, AUTOTUTORS in AutoDockTools was used to define all possible flexible torsions of selected ligands to favor the computed binding with the receptor structure [62]. Caspase-3 (PDB ID: 5I9B), Caspase-7 (PDB ID: 1f1j), MMP-7 (PDB ID: 2Y6D), and p53 (PDB ID: 1TSR) crystal structures were downloaded from the Protein Data Bank (https://rcsb.org; accessed on 2 March 2022), and all bonded ligands, ions, and solvent molecules were manually removed using the DS Visualizer 2.5 program. For the docking studies, the structure of selected proteins was parameterized using AutoDock Tools [63]. In order to facilitate the formation of hydrogen bonds, polar hydrogens were added. AutoDock Vina software was used to perform molecular docking and default procedures for docking a flexible ligand to a rigid protein. Then, ligands were centered at the binding site located into the binding cavity of the caspase-3, (x = 1.5, y = −8.1, z = −13.4), Caspase-7 (x = 37.955, y = 23.117, z = −2.671), MMP-7 (x = −18.192, y = 9.495, z = 2.408) and p53 (x = 56.907, y = 28.404, z = 85.947). In detail, docking studies involved a grid box which was identified by using Autodock Vina 1.1.2 (accessed on 3 March 2022), and exhaustiveness was 20 for each protein-compound pair [63]. Catalytic active site was surrounded by a docking grid of 40 × 40 × 40 Å (for caspase-3), 42 × 42 × 42 Å (for caspase-7), 40 × 40 × 40 Å (for MMP-7), and 32 × 32 × 32 Å (for p53) with a grid spacing of 1 Å. Ligand-binding affinities (in kcal/mol) were estimated by AutoDock Vina and ranked based on the free-energy binding theory. Then, docking solutions were graphically inspected by using the DS Visualizer 2.5 (http://3dsbiovia.com/products/; accessed on 5 March 2022) to provide a 2D-ligand interaction plot, while ribbon surface representation of 3D model was explored by using The PyMOL Molecular Graphics System Version 2.0 Schrodinger, LLC (2015).

## 4. Conclusions

Different investigations have shown the importance of using hybrid molecules as potential therapeutic agents for various maladies. Compounds based on curcumin and resveratrol have emerged as potential candidates to treat CRC due to the wide spectrum of biological activities. In this investigation, hybrid molecules **3a, 3e, 3i**, and **3k** induced important changes in mitochondrial membrane potential of SW620 and SW480 cells, with considerable loss in membrane integrity, suggesting a death process. We propose that compounds **3i** on SW480 and **3d** on SW620 induce apoptosis mediated by caspases but independent of the p53 status. In addition, considering that hybrids **3e, 3i**, and **3k** caused G0/G1 phase arrest could imply that these molecules act as cytotoxic and cytostatic compounds, making them potential therapeutic alternatives or adjuvants in the treatment of colorectal cancer, even in those cases with resistance to conventional chemotherapy because of the lack of p53 expression or function. Docking studies suggest that targeting proapoptotic human caspases 3/7 proteins, as well as human MMP-7 and p53, could be responsible for those effects observed in vitro in the cytotoxic response of **3e**, **3i**, and **3k** in SW480 and SW620 colon carcinoma cell lines; however, complementary studies are needed to elucidate the exact mechanism of action associated with these hybrid molecules.

## Figures and Tables

**Table 1:** Best binding energy (kcal/mol) based on AutoDock scoring of the hybrids **3e, 3i** and **3k** against multiples molecular targets.

Target Protein	Ligands (Docking Score, kcal/mol)						
	3e	3i	3k	HLI ^a^	Ac-DEVD-CMK ^b^	NSC194598 ^c^	SCH529074 ^d^
Human MMP-7	−10.4	−10.2	-	−8.3	-	-	-
Caspase 3	-	−8.5	−8.3	-	−8.2	-	-
Caspase 7	-	−7.8	-	-	−8.2	-	-
Mutant p53	−7.3	-	-	-	-	−7.3	−7.0

^a^ MMP-7 hydroxamate ligand inhibitor; ^b^ Potent cell-permeable and irreversible inhibitor of caspase-3/7; ^c^ Quinazolin-based p53-DNA-binding inhibitor with in vitro IC_50_ of 180 nM [55]; ^d^ Potent and orally p53-DNA noncompetitive activator of mutant p53, binding to p53 DNA binding domain (IC_50_ = 1–2 µM, in vitro) [56].

## Data Availability

Not applicable.
